# SAMHD1 Regulates Human Papillomavirus 16-Induced Cell Proliferation and Viral Replication during Differentiation of Keratinocytes

**DOI:** 10.1128/mSphere.00448-19

**Published:** 2019-08-07

**Authors:** Claire D. James, Apurva T. Prabhakar, Raymonde Otoa, Michael R. Evans, Xu Wang, Molly L. Bristol, Kun Zhang, Renfeng Li, Iain M. Morgan

**Affiliations:** a Philips Institute for Oral Health Research, School of Dentistry, Virginia Commonwealth University (VCU), Richmond, Virginia, USA; b VCU Massey Cancer Center, Richmond, Virginia, USA; Northwestern University

**Keywords:** SAMHD1, differentiation, host-pathogen interactions, life cycle, papillomavirus, viral replication

## Abstract

Human papillomaviruses induce a host of anogenital cancers, as well as oropharyngeal cancer (HPV+OPC); human papillomavirus 16 (HPV16) is causative in around 90% of HPV+OPC cases. Using telomerase reverse transcriptase (TERT) immortalized foreskin keratinocytes (N/Tert-1), we have identified significant host gene reprogramming by HPV16 (N/Tert-1+HPV16) and demonstrated that N/Tert-1+HPV16 support late stages of the viral life cycle. Expression of the cellular dNTPase and homologous recombination factor sterile alpha motif and histidine-aspartic domain HD-containing protein 1 (SAMHD1) is transcriptionally regulated by HPV16 in N/Tert-1. CRISPR/Cas9 removal of SAMHD1 from N/Tert-1 and N/Tert-1+HPV16 demonstrates that SAMHD1 controls cell proliferation of N/Tert-1 only in the presence of HPV16; the deletion of SAMHD1 promotes hyperproliferation of N/Tert-1+HPV16 cells in organotypic raft cultures but has no effect on N/Tert-1. Viral replication is also elevated in the absence of SAMHD1. This new system has allowed us to identify a specific interaction between SAMHD1 and HPV16 that regulates host cell proliferation and viral replication; such studies are problematic in nonimmortalized primary keratinocytes due to their limited life span. To confirm the relevance of our results, we repeated the analysis with human tonsil keratinocytes (HTK) immortalized by HPV16 (HTK+HPV16) and observed the same hyperproliferative phenotype following CRISPR/Cas9 editing of SAMHD1. Identical results were obtained with three independent CRISPR/Cas9 guide RNAs. The isogenic pairing of N/Tert-1 with N/Tert-1+HPV16, combined with HTK+HPV16, presents a unique system to identify host genes whose products functionally interact with HPV16 to regulate host cellular growth in keratinocytes.

**IMPORTANCE** HPVs are causative agents in human cancers and are responsible for around of 5% of all cancers. A better understanding of the viral life cycle in keratinocytes will facilitate the development of novel therapeutics to combat HPV-positive cancers. Here, we present a unique keratinocyte model to identify host proteins that specifically interact with HPV16. Using this system, we report that a cellular gene, SAMHD1, is regulated by HPV16 at the RNA and protein levels in keratinocytes. Elimination of SAMHD1 from these cells using CRISPR/Cas9 editing promotes enhanced cellular proliferation by HPV16 in keratinocytes and elevated viral replication but not in keratinocytes that do not have HPV16. Our study demonstrates a specific intricate interplay between HPV16 and SAMHD1 during the viral life cycle and establishes a unique model system to assist exploring host factors critical for HPV pathogenesis.

## Abstract (precis)

HPVs are causative agents in human cancers and are responsible for around of 5% of all cancers. A better understanding of the viral life cycle in keratinocytes will facilitate the development of novel therapeutics to combat HPV-positive cancers. Here, we present a unique keratinocyte model to identify host proteins that specifically interact with HPV16. Using this system, we report that a cellular gene, SAMHD1, is regulated by HPV16 at the RNA and protein levels in keratinocytes. Elimination of SAMHD1 from these cells using CRISPR/Cas9 editing promotes enhanced cellular proliferation by HPV16 in keratinocytes and elevated viral replication but not in keratinocytes that do not have HPV16. Our study demonstrates a specific intricate interplay between HPV16 and SAMHD1 during the viral life cycle and establishes a unique model system to assist exploring host factors critical for HPV pathogenesis.

## INTRODUCTION

High-risk human papillomaviruses (HPVs) are the etiologic agents of a number of cancers, including anogenital and oropharyngeal carcinomas (OPC) (1). Upwards of 70% of new OPC cases per year are HPV positive, with the high-risk type 16 responsible for 90% of these HPV-positive cases (2). There have been extensive studies investigating the HPV16 life cycle in anogenital keratinocytes immortalized by the virus (see, for example, references 3–6). Such studies have been crucial in enhancing our understanding of the viral life cycle and identifying novel therapeutic targets that could be targeted for the alleviation of HPV-induced cancers and/or disruption of the viral life cycle. Recently, we developed a system for looking at the HPV16 life cycle in TERT immortalized keratinocytes. This was done by introducing the HPV16 genome into TERT immortalized foreskin keratinocytes (N/Tert-1+HPV16) and carrying out organotypic raft cultures followed by confirmation of late stages of the viral life cycle, including E1^E4 and E2 expression, as well as amplification of the HPV16 genome in the differentiated epithelial cells. The utility of this model is that we retain isogenic parental cells that can be analyzed and genetically manipulated side by side with the virus-encoding cells. We used this model to determine host reprogramming induced by HPV16 in keratinocytes and a large number of innate immune genes were shown to be downregulated, as others have demonstrated before (7). One of the innate immune genes predicted to be downregulated was sterile alpha motif and histidine-aspartic domain HD-containing protein 1 (SAMHD1). To our knowledge, this is the first time that downregulation of SAMHD1 expression by HPV16 has been reported.

SAMHD1 is a deoxynucleotide triphosphate triphosphohydrolase (dNTPase) enzyme, which regulates intracellular levels of dinucleotide triphosphates (dNTPs) and acts as an intrinsic immune response factor (8, 9). To function as a dNTPase, the protein forms a homotetramer, which is destabilized by phosphorylation. Each individual protein is comprised of two domains, a sterile alpha motif (SAM), which mediates protein-protein contacts often with other SAM domains, and a dGTP-regulated dNTP hydrolase domain (HD), which decreases cellular dNTP levels (10–12). The HD domain has also been shown to be required for protein oligomerization and RNA binding and has been suggested to have nuclease activity, although this is a matter of debate (13–15).

Our interest in SAMHD1 was stimulated by the other known restriction roles this protein has in viral life cycles; SAMHD1 has been predominantly characterized as a host restriction factor for human immunodeficiency virus (HIV) (15, 16). The inhibitory mechanism against HIV type 1 (HIV-1) was first linked to the dNTPase activity of SAMHD1, which lowers the intracellular dNTP concentration below the level required for viral reverse transcription. It has been suggested that SAMHD1, or associated proteins TREX1 and RNaseH1, restricts HIV-1 through an RNase activity (13). However, the relative contribution of these two activities in viral restriction is still a matter of debate, and some reports challenge the presence of nuclease activity (14–16). In cycling cells, SAMHD1 is phosphorylated by cyclin-dependent kinase 1 or 2 (CDK1 or CDK2, respectively) at threonine 592 (16). Dephosphorylation at residue T592 regulates the resistance to HIV-1 infection in noncycling cells, such as myeloid cells or resting T cells (16, 17). However, whether the phosphorylation of SAMHD1 at residue T592 influences the enzymatic function of the protein remains controversial; it has been suggested that this residue does not influence the dNTPase function (18), whereas other studies propose that dNTPase-competent SAMHD1 homotetramers are destabilized through phosphorylation at T592 (19). Therefore, the precise role for T592 phosphorylation in regulating SAMHD1 function remains to be fully elucidated.

SAMHD1 is an interferon-stimulated gene (ISG). SAMHD1 function is elevated by interferon, suggesting that it could be involved broadly in antiviral defense (20). In support of this idea, SAMHD1 not only restricts HIV but also DNA viruses (21–23). There is also evidence of viruses countering SAMHD1 restrictive activity. The HIV-2 encoded accessory factor Vpx, which is also found in closely related simian immunodeficiency virus (SIV) strains, degrades SAMHD1 through a proteasome-dependent mechanism (24). Our recent study has also shown that conserved herpesvirus protein kinases antagonize SAMHD1 restriction through phosphorylation (25). In addition, SAMHD1 has recently been shown to regulate the human cytomegalovirus life cycle (26). Downregulation of SAMHD1 expression by HPV16 could also counter the antiviral activity of this protein.

In this report, we confirm that SAMHD1 RNA and protein levels are downregulated by HPV16 in N/Tert-1 cells. Overexpression of SAMHD1 had no effect on the HPV16 viral life cycle in keratinocytes, but it was notable that the exogenous SAMHD1 was not expressed in the differentiated epithelium; therefore, it could not have any effect on viral genome amplification which occurs in the differentiated epithelium. CRISPR/Cas9 editing of SAMHD1 resulted in hyperproliferation of basal cells during organotypic raft cultures of N/Tert-1 containing HPV16 but not in parental N/Tert-1. This was confirmed by a “thickening” of the epithelium by HPV16 in the absence of SAMHD1, by elevated bromodeoxyuridine (BrdU) labeling of basal cells, and enhanced expression of the S-phase marker protein cyclin E. There was also increased viral replication in the differentiated layer of the epithelium in the absence of SAMHD1. These results were obtained using 3 independent guide RNAs for CRISPR/Cas9 editing, and also in tonsil keratinocytes immortalized by HPV16. In proliferating monolayer cells CRISPR/Cas9 targeting of SAMHD1 had no effect on either cell proliferation of viral genome copy number. Therefore, the results present an intricate interplay between the virus and SAMHD1 that contributes to a controlled HPV16 life cycle only in differentiating epithelium. The results also demonstrate the utility of our N/Tert-1 system, as it has allowed us to detect hyperproliferation of epithelial cells in the absence of SAMHD1 only in the presence of HPV16, demonstrating a functional interaction between HPV16 and SAMHD1 that regulates cellular growth. This would be technically limiting using primary keratinocytes, as it would be difficult to generate primary cells with SAMHD1 knocked out that would retain any proliferative capacity for differentiation studies. We propose that our system offers a unique model for identifying host proteins that specifically interact with HPV16 to regulate host cell growth and viral replication in keratinocytes.

## RESULTS

### SAMHD1 is downregulated in HPV16 containing keratinocytes.

**FIG 1:**
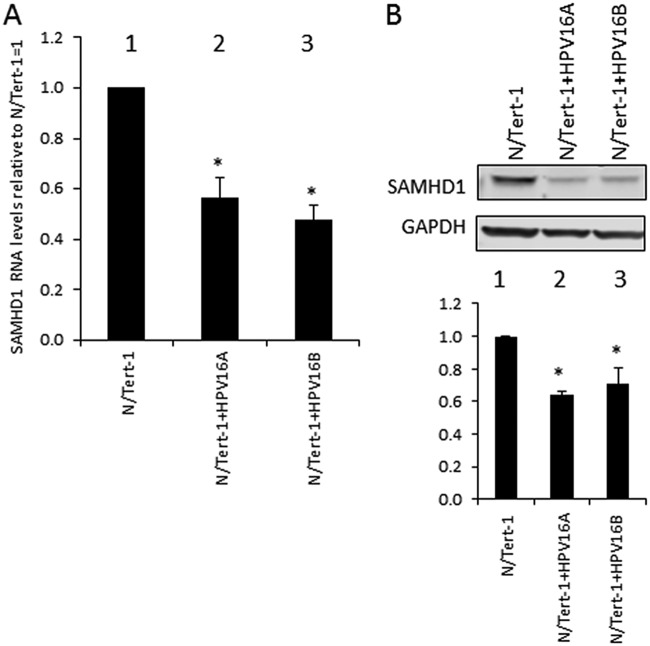
SAMHD1 is downregulated in HPV16-positive keratinocytes. (A) RNA expression levels of SAMHD1 in N/Tert-1 cells (lane 1) and HPV16-containing N/Tert-1 cells (lanes 2 and 3). Results are expressed as fold change from that observed in parental N/Tert-1 cells and represent the averages from three independent experiments. (B) Western blot analysis was carried out on protein extracted from the N/Tert-1 cells (lane 1) and HPV16-containing N/Tert-1 cells (lanes 2 and 3). GAPDH is shown as an internal control. Western blots were visualized and quantitated using a Li-Cor system and calculated relative to parental N/Tert-1. Data in panels A and B represent the averages of 3 independent experiments, and error bars indicate standard error of the mean. *, *P* < 0.05.

Previous work from this lab developed and characterized an HPV16 life cycle model in N/Tert-1 cells (7). Initial comparison of two clonal cell lines containing HPV16 that support late stages of the viral life cycle (N/Tert-1+HPV16A and N/Tert-1+HPV16B) with the parental N/Tert-1 revealed a decrease in both SAMHD1 RNA and protein expression (Fig. 1A and B, respectively). In the presence of HPV16, SAMHD1 RNA is expressed at a 50% lower level than the N/Tert-1 (Fig. 1A), which validates our previous observations from RNA sequencing (RNA-seq) analysis (7). Protein levels are decreased correspondingly (Fig. 1B). This was consistent in three individual repeats, which were quantified (Fig. 1B).

### Downregulation of SAMHD1 by HPV16 is maintained during differentiation.

**FIG 2:**
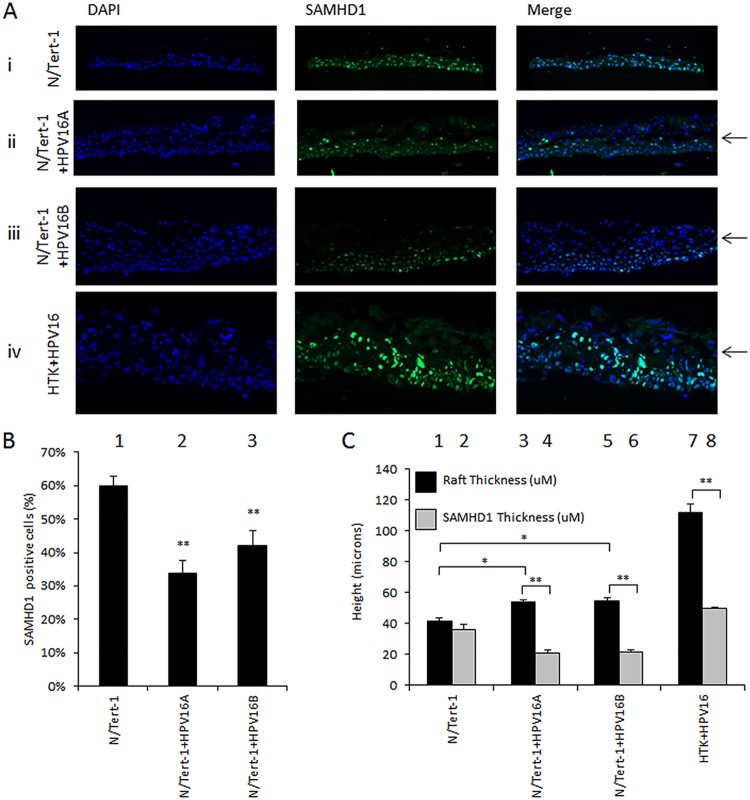
SAMHD1 is downregulated by HPV16 in differentiated epithelia. (A) N/Tert-1 (i), N/Tert-1+HPV16A (ii), N/Tert-1+HPV16B (iii), and human tonsil keratinocytes immortalized by HPV16 (HTK+HPV16) (iv) were differentiated in culture, and the resulting sections were stained for SAMHD1. Arrows indicate the level at which SAMHD1 disappears. (B) SAMHD1-positive cells of three sections from two individual rafts were quantified computationally and the measurements averaged. Immunofluorescence was quantified using a Vectra Polaris automated imaging system, where whole stained sections were scanned computationally and the intensity calculated compared to a negative background control (secondary antibody only) and a positive localization control (DAPI). The same imaging parameters were used for each slide. For each sample, two sections from three individual rafts were scanned to generate average values shown in this graph. Error bars indicate standard error of the mean. *, *P* < 0.05. (C) The overall thickness and region of SAMHD1 positivity from three sections from two individual rafts were quantified computationally using a Vectra Polaris automated imaging system and the measurements averaged. Error bars indicate standard error of the mean. *, *P* < 0.05.

In order to assess the expression of SAMHD1 during the HPV16 life cycle, N/Tert-1 and N/Tert-1+HPV16 were differentiated by organotypic “raft” culture. The rafts were then fixed and subject to immunofluorescent staining to determine SAMHD1 levels and localization in differentiated epithelia. SAMHD1 is expressed in N/Tert-1 throughout the organotypic section (Fig. 2A), whereas fewer cells are stained when HPV16 is present (N/Tert-1+HPV16A and N/Tert-1+HPV16B). Three independent organotypic raft cultures were stained and SAMHD1 levels quantitated using a Vectra Polaris automated imaging system; the difference in staining between HPV-negative and -positive N/Tert-1 cells is significant (Fig. 2B). Furthermore, the presence of HPV16 in N/Tert-1 leads to the loss of SAMHD1 expression in the upper layers of the epithelium (Fig. 2C). This was quantified by measuring the “height” to which SAMHD1 is expressed in the rafts using a Vectra Polaris automated imaging system; in the case of HPV16-positive N/Tert-1 raft sections, SAMHD1 expression occurs from the basal layer to halfway up the raft, while in N/Tert-1, staining is observed throughout the differentiated epithelium. It is also clear that the intensity of the SAMHD1 staining is diminished in the presence of HPV16 in N/Tert-1 (Fig. 2A). Human tonsil keratinocytes immortalized by HPV16 (HTK+HPV16 in Fig. 2A and C) were also stained for SAMHD1, and again, it is clear that in the upper layers of the differentiated epithelium, there are no detectable levels of SAMHD1 staining (Fig. 2A and C). These results confirm that the expression of SAMHD1 is downregulated by HPV16 during the viral life cycle in differentiating keratinocytes containing HPV16.

### Deletion of SAMHD1 results in hyperproliferation of HPV16-positive keratinocytes in organotypic raft cultures.

**FIG 3:**
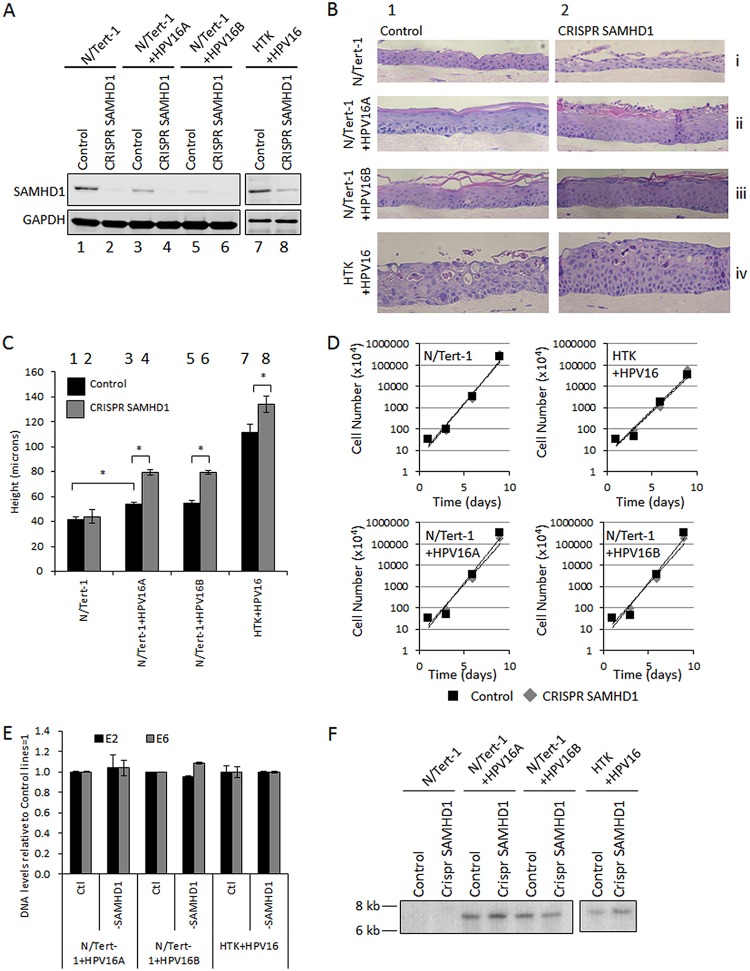
Generation and preliminary analysis of SAMHD1 CRISPR N/Tert-1 cells. (A) SAMHD1 knockdown cell lines were generated in N/Tert-1, N/Tert-1+HPV16A, and HTK+HPV16 cells by infection of cells with lentiviruses containing SAMHD1 CRISPR guide RNAs, followed by selection with puromycin. Depletion of SAMHD1 was confirmed by Western blotting; samples from HTK+HPV16 (lanes 7 and 8) were run on a separate gel. Control cells were generated identically but with control guide RNA. (B) Representative images of rafts that were quantified. Compared to parental lines (column 1), SAMHD1 CRISPR lines (column 2) appeared to be hyperproliferative where HPV16 was present. (C) Three H&E-stained sections from two individual rafts were imaged, and measurements were taken at 100-μm intervals across each section using a Vectra Polaris automated imaging system and quantitated. Error bars indicate standard error of the mean. *, *P* < 0.05. (D) SAMHD1 CRISPR cell lines were plated alongside parental cell lines (3 × 10^5^) grown for 3 days before harvesting and counting. This was repeated three times to assess cell growth rates in monolayer grown cells, which are unaltered by the depletion of SAMHD1. (E) Southern blots were carried out on DNA extracted from the indicated cell lines. The DNA was digested with Sph1 to linearize the 8-kbp viral genome, and the resultant blot was probed with the labeled HPV16 DNA. (F) DNA was extracted from control and SAMHD1 CRISPR cell lines and subject to PCR detection of HPV16 E2 and HPV16 E6. DNA from N/Tert-1 cells was utilized as a negative control. Triplicate samples were analyzed and averaged, and error bars indicate the standard error of the mean.

While HPV16 downregulates the expression levels of SAMHD1 in keratinocytes (Fig. 1 and 2), there remains a substantial level of SAMHD1 in the HPV16-positive cells. To determine whether this remaining SAMHD1 played an important role in regulating the HPV16 life cycle in keratinocytes, SAMHD1 expression was removed using CRISPR/Cas9 targeting. Figure 3A demonstrates successful downregulation of SAMHD1 protein in the targeted cells. These were pools of cells; therefore, a residual level of SAMHD1 remains in cells not successfully targeted by CRISPR/Cas9. Having established stable knockdowns, these cells were differentiated via organotypic raft culture. Initial hematoxylin and eosin (H&E) staining revealed a hyperproliferative phenotype in epithelia where SAMHD1 was knocked down and HPV16 was present, compared to control cells (N/Tert-1 cells by themselves) (Fig. 3B). Triplicate rafts were sectioned and stained with H&E before measurement using a Vectra Polaris automated imaging system. Quantification revealed that this increase in section thickness by HPV16 was significant (Fig. 3C). This hyperproliferation was not due to the SAMHD1-depleted cell lines growing quicker in monolayer cells (Fig. 3D). Additionally, there was no significant difference in HPV16 genome copy number in monolayer cells that underexpress SAMHD1 (Fig. 3E and F).

**FIG 4:**
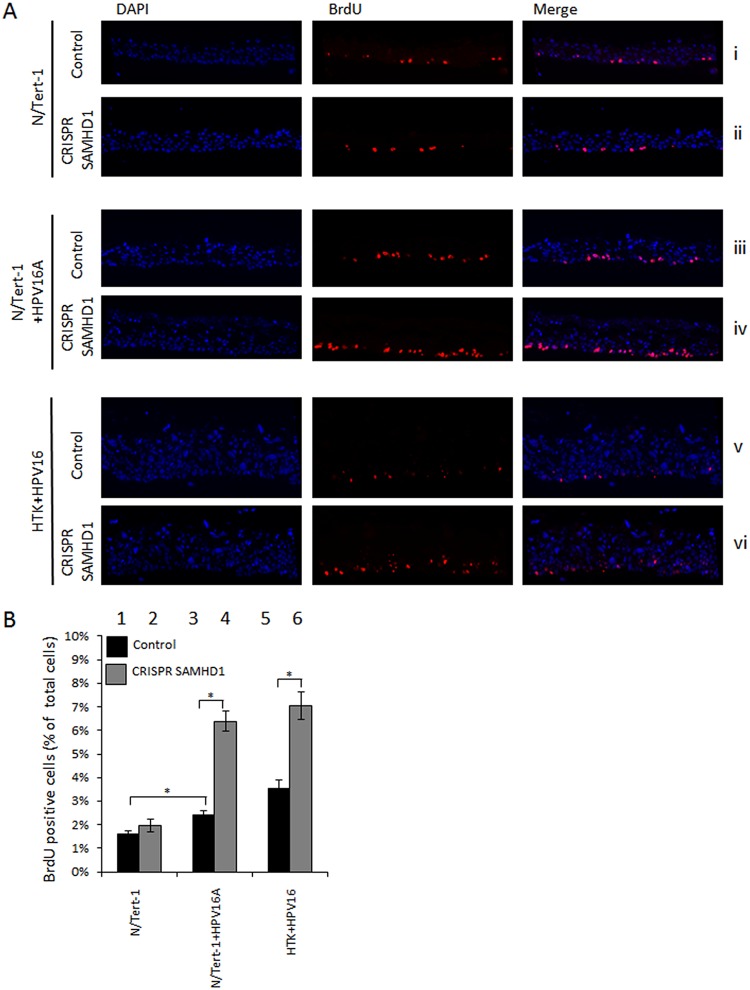
Knockdown of SAMHD1 increases BrdU incorporation in basal cells of HPV16-positive epithelia. (A) The nucleoside analog BrdU was included in media for the final 16 h of organotypic raft culture. Fixed sections were then stained for BrdU incorporation and for differentiation marker involucrin. (B) The number of BrdU-positive cells was measured using a Vectra Polaris imaging system, whereby whole stained sections were scanned computationally and the intensity calculated compared to a negative background control (secondary antibody only) and a positive localization control (DAPI). The same imaging parameters were used for each slide. Three sections from two individual rafts were subject to analysis, and error bars indicate standard error of the mean. *, *P* < 0.05.

To investigate whether this hyperproliferation was specific to a certain layer of the differentiated culture, rafts were treated with BrdU for the final 16 h of culture before being fixed and sectioned. BrdU staining highlights the cells actively dividing in those 16 h. In N/Tert-1 cells, there was no increase in BrdU-positive cells in the absence of SAMHD1 (Fig. 4A). In N/Tert-1+HPV16 (A and B clones) cells, there was an increase in BrdU labeling compared with N/Tert-1 cells, demonstrating the expected enhanced proliferation induced by HPV16 (Fig. 4A). In addition, when SAMHD1 was removed, there was a further increase in BrdU labeling in the presence of HPV16 in the basal layer that is not observed in the parental N/Tert-1 cells. These experiments were repeated with HTK+HPV16 cells lacking SAMHD1 (Fig. 4Av and vi), where, as with N/Tert-1+HPV16, there is an increase in BrdU-positive cells in the basal layer in the absence of SAMHD1. These experiments were repeated and quantitated using a Vectra Polaris automated imaging system (Fig. 4B), and there is a statistically significant increased level of BrdU-positive cells in N/Tert-1+HPV16 and HTK+HPV16 but not in N/Tert-1 cells. This demonstrates an interaction between HPV16 and SAMHD1 that controls cellular proliferation in the basal layers of the epithelium.

**FIG 5:**
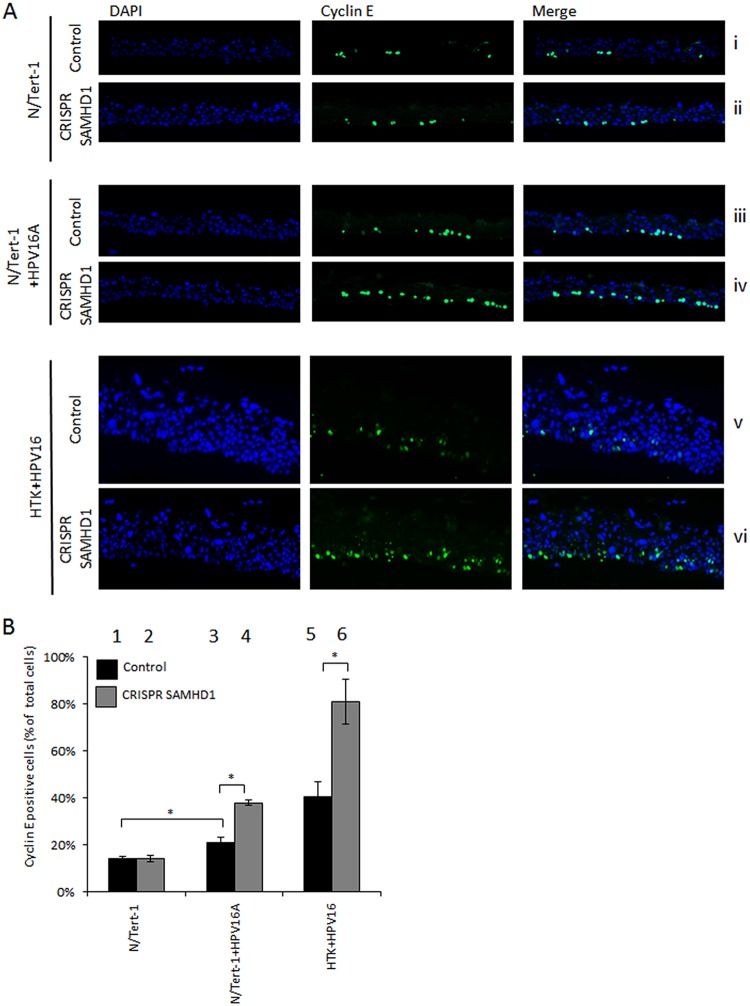
Knockdown of SAMHD1 increases cyclin E staining in basal cells of HPV16-positive epithelia. (A) Differentiated sections from organotypic raft cultures were stained for S-phase marker cyclin E. Representative images of cyclin E staining in differentiated N/Tert-1, N/Tert-1+HPV16, and HTK+HPV16 cell lines are shown. (B) The number of cyclin E-positive cells was counted was measured using the Vectra Polaris imaging system, whereby whole stained sections were scanned computationally and the intensity calculated compared to a negative background control (secondary antibody only) and a positive localization control (DAPI), using the same imaging parameters for each slide. Three sections from two individual rafts were subject to analysis, and the error bars indicate the standard error of the mean. *, *P* < 0.05.

To further confirm the proliferative nature of these cells, cells were stained with cyclin E, a protein expressed in S phase (27). In N/Tert-1 cells, there is no increase in cyclin E staining without SAMHD1 (Fig. 5Ai and ii), while there is increased cyclin E staining in the absence of SAMHD1 in N/Tert-1+HPV16A (Fig. 5Aiii and iv). This increase in cyclin E staining in the absence of SAMHD1 was also observed in HTK+HPV16 cells lacking SAMHD1 compared with parental HTK+HPV16 (Fig. 5Av and vi). These results were repeated and cyclin E-positive cells quantitated using a Vectra Polaris automated imaging system, and a summary of the results is presented graphically in Fig. 5B. In N/Tert-1+HPV16 and HTK+HPV16 cells, there is a statistically significant increase in cyclin E expression in the absence of SAMHD1, while no such increase is observed in N/Tert-1 cells.

**FIG 6:**
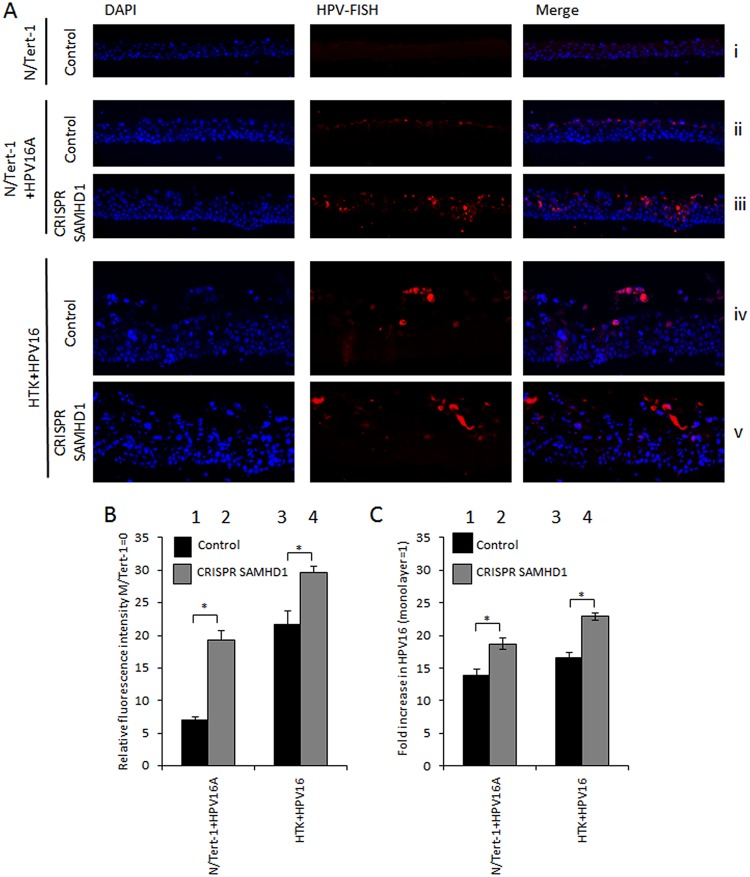
Knockdown of SAMHD1 increases HPV16 amplification in differentiated culture. (A) N/Tert-1+HPV16A and HTK+HPV16 cells were differentiated in culture, formalin fixed, paraffin embedded, sectioned, and then stained HPV16 genomes using DNA-FISH. Images shown are representative images of HPV-FISH in differentiated culture. (B) The intensity of fluorescence was quantified using the Vectra Polaris imaging system, whereby whole sections were scanned computationally and the intensity calculated compared to a negative background control (N/Tert-1). (C) Amplification of HPV16 DNA was measured by qPCR. DNA was extracted from monolayer or raft samples using HIRT buffer and subject to SYBR green qPCR using HPV16 E2 primers. Threshold cycle (*C_T_*) values were normalized to mitochondrial DNA (Δ*C_T_*) and then rafted to corresponding monolayer (ΔΔ*C_T_*) to define the fold increase in HPV16 DNA. Error bars indicate standard error of the mean. *, *P* < 0.05.

As there was a hyperproliferation of the HPV16-positive cells in the absence of SAMHD1 and an increased thickening of the differentiated epithelium, we investigated whether there was an enhanced amplification of the HPV16 genome in the cells lacking SAMHD1. To do this, fluorescence *in situ* hybridization (FISH) analysis for the viral DNA was carried out, and the intensity of the staining was measured using the Vectra Polaris automated imaging system; whole stained sections were scanned computationally and the intensity and localization of staining measured relative to a negative control (N/Tert-1) and a positive control (N/Tert-1+HPV16A). Figure 6A shows representative images from the staining. Figure 6B summarizes the results of these experiments, and when SAMHD1 is removed, there is a statistically significant enhanced FISH signal detected, indicating increased HPV16 replication. In Fig. 6Ai, it is clear that there is no signal in the N/Tert-1, while in Fig. 6Aii and iii, there is an enhanced FISH signal in the absence of SAMHD1. In HTK+HPV16 cells (Fig. 6Aiv), there is also an increase in signal. Please note that the measurement of the signal is quantitative and nonsubjective, while the images are representative. There is also an increase in viral genome detection throughout the differentiated tissue in the absence of SAMHD1. To quantitate this difference in HPV16 replication, we rafted cells and extracted DNA from them. We carried out quantitative PCR (qPCR) and compared the signal with that obtained in the monolayer cultures of N/Tert-1+HPV16A and HTK+HPV16 cells (Fig. 6C). There is a significant increase in viral genome copy number following differentiation (the DNA was standardized to the small circular mitochondrial DNA genome) in both N/Tert-1+HPV16 and HTK+HPV16 cells. In the absence of SAMHD1, there was a further increase in the DNA signal compared with corresponding monolayer cells, demonstrating that there is more viral replication in the absence of SAMHD1 during epithelial differentiation.

**FIG 7:**
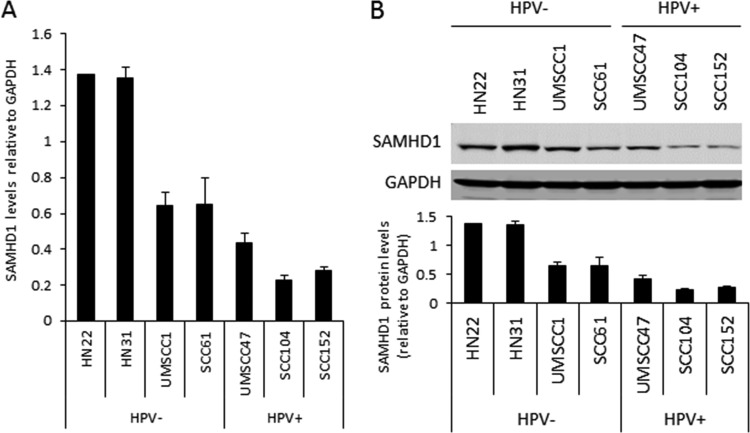
Decreased levels of SAMHD1 correlate with HPV16 in HNSCC-derived cell lines. (A) RNA expression levels of SAMHD1 in HPV-negative (lanes 1 to 4) and HPV-positive (lanes 5 to 7) HNSCC cell lines. Results are expressed as the fold change (Δ*C_T_*) relative to internal control (GAPDH) averages from three independent experiments. (B) Western blot analysis was carried out on protein extracted from the HPV-negative (lanes 1 to 4) and HPV-positive (lanes 5 to 7) HNSCC cell lines. GAPDH is shown as an internal control. Western blots were visualized and quantified using Li-Cor system and calculated relative to GAPDH. Data in A and B represent the averages from 3 independent experiments, and error bars indicate the standard error of the mean.

We investigated the expression levels of SAMHD1 at the RNA and protein levels in a panel of HPV16-negative and -positive head and neck cancer cell lines (Fig. 7). At the RNA level, the three HPV16-positive lines had the lowest levels of SAMHD1 RNA (Fig. 7A), and the RNA level was reflected in the protein expression (Fig. 7B). Therefore, it is possible that this downregulation of SAMHD1 by HPV16 in keratinocytes persists throughout the transformation process through to HPV16-positive cancer cells.

Finally, we reproduced our results with an additional two SAMHD1 guide RNAs that target different sequences in the SAMHD1 gene. Figure S1A in the supplemental material details the knockdown of SAMHD1 expression by these additional CRISPR/Cas9 targeting sequences. Figure S1B demonstrates a thickening of the epithelium in N/Tert-1 cells only in the presence of HPV16 following SAMHD1 removal, while Fig. S1C and D demonstrate the enhanced BrdU and cyclin E staining, respectively, when SAMHD1 is removed in the presence of HPV16. Overall, the results with the three independent SAMHD1 guide RNAs are identical, demonstrating that the results are not due to off-target effects of the SAMHD1 guide RNAs.

10.1128/mSphere.00448-19.1 FIG S1 Summary of results with two additional SAMHD1 guide RNAs. Download FIG S1, TIF file, 2.8 MB. Copyright © 2019 James et al. 2019 James et al. This content is distributed under the terms of the Creative Commons Attribution 4.0 International license.

## DISCUSSION

Many excellent studies have been carried out with primary human keratinocytes to characterize the immortalization properties of high-risk HPV (HR-HPV). These HPV immortalized cells have proved invaluable at identifying cellular proteins that are required for the life cycle of HR-HPV (3–6, 28–42). For example, it is clear that host cell homologous recombination factors are required for the amplification phase of the viral life cycle (5, 38, 39). However, there are inevitably some limitations to using HPV immortalized keratinocytes. For example, if genes are knocked down to investigate the role in the viral life cycle, it is difficult to investigate what the consequences of this knockdown are in non-HPV immortalized cells. This is because selection of primary cells with host genes knocked down takes several cell passages, and primary cells will often senesce prior to selection. Even if knockdown cells were generated, they would have a very limited life span for making organotypic rafts to investigate the consequences of the gene knockdown on normal cell differentiation. We wished to generate an isogenic system that would allow us to carry out CRISPR/Cas9 editing in parental cells and compare them with cells containing HPV16. To do this, we generated clonal cell lines of N/Tert-1 that retain episomal HPV16 genomes and demonstrated transcriptional reprogramming by HPV16 in these cells that is related to that regulated in HPV16-positive head and neck cancers. These N/Tert-1+HPV16 cells, upon organotypic raft culture, demonstrate several markers of the late stages of the viral life cycle, including E1^E4 expression, E2 expression, and amplification of the viral genome in the upper layers of the differentiated epithelium (7). Importantly, in our studies using this system, we have validated gene expression changes in human tonsil keratinocytes immortalized by HPV16 (HTK+HPV16) and also in HPV16-positive head and neck cancers.

Here, we report the utility of this system. We demonstrate that the removal of SAMHD1 from N/Tert-1+HPV16 and HTK+HPV16 cells results in hyperproliferation of these cells in organotypic raft cultures. This increase in proliferation was confirmed by a “thickening” of the raft culture and an increase in BrdU and cyclin E-positive cells in the absence of SAMHD1 (both S-phase markers). We also observed an elevation in viral genome amplification in the absence of SAMHD1. The parental N/Tert-1 cells exhibit no increase in BrdU- or cyclin E-positive cells, demonstrating that the increased proliferation was due to the presence of HPV16. If we had carried out these studies in the absence of the N/Tert-1 cells, we would not have been able to determine whether the deletion of SAMHD1 expression by itself was proliferative for the cells. Therefore, this system has allowed us to identify an interaction between SAMHD1 and HPV16 that regulates the proliferation of N/Tert-1+HPV16 and HTK+HPV16 cells. The addition of HTK+HPV16 demonstrates that the SAMHD1 deletion phenotype is retained in cell lines immortalized by HPV16. Therefore, the combination of N/Tert-1, N/Tert-1+HPV16, and HTK+HPV16 represents an excellent system for identifying interactions between host proteins and HPV16 that have an effect on host cell growth and viral replication.

SAMHD1 is a dNTPase, and therefore, HPV16 downregulation of this protein would potentially enhance the dNTP pool available in the cell and therefore boost HPV16 replication, particularly in the upper layers of the differentiating epithelium where viral genome amplification occurs. It is striking that the virus eliminates detectable SAMHD1 expression in the upper layers of the differentiating epithelium where viral genome amplification occurs (Fig. 2). SAMHD1 is also a homologous recombination (HR) factor and is involved in recruiting MRE11 to damaged DNA (43, 44). This role of SAMHD1 could also play an important role in its interaction with HPV16, as this virus recruits a host of HR factors to its replicating DNA, and it is proposed that HPV16 uses HR during viral replication in order to amplify its genome. However, unlike downregulation of other HR factors (5, 38, 39), downregulation of SAMHD1 boosts viral genome amplification and also disrupts the equilibrium between the virus and the host cell.

The reason for the hyperproliferation of HPV16-containing keratinocytes in the absence of SAMHD1 is not clear. This is not observed in monolayer cultures and therefore only becomes apparent during organotypic raft cultures, indicating that there may be some involvement of the collagen-fibroblast plug used to generate the differentiating cells. This collagen-fibroblast plug could mimic a stroma-epithelial cell interaction, and there is a known cross talk between HPV and the stroma (45). It is also apparent that there is not an increase in HPV16 genomes in the absence of SAMHD1 in monolayer cells, so the difference is not due to an initial increased viral genome copy number in these cells upon rafting.

SAMHD1 is a restriction factor for HIV and other DNA viruses, including hepatitis B virus (HBV) (23), herpes simplex virus 1 (HSV-1) (21), Epstein-Barr virus (EBV) (25), and cytomegalovirus (CMV) (26), and here, we demonstrate that SAMHD1 is also a restriction factor for HPV16. Not only does the absence of SAMHD1 promote hyperproliferation of the infected cells, it also allows an enhanced amplification of the HPV16 genome during differentiation. In addition, the FISH staining for the HPV16 genome in Fig. 6 suggests that in N/Tert-1+HPV16 and HTK+HPV16 cells in the absence of SAMHD1, there is an increase in viral signal in the lower layers of the epithelium. This is hard to quantitate but does suggest that during differentiation, the increased replication of the viral genome perhaps starts early in the differentiation process.

Our isogenic N/Tert-1 system for investigating the HPV16 life cycle has been essential at revealing a specific interaction between the virus and SAMHD1 that controls host cell proliferation. In addition, the enhanced genome amplification in the absence of SAMHD1 demonstrates that SAMHD1 is a restriction factor for HPV16. The virus clearly downregulates SAMHD1 expression but retains a level that is required for controlling both host proliferation and viral genome amplification. Perhaps, downregulation of SAMHD1 is required to generate an enhanced pool of nucleotides that would promote viral genome amplification but SAMHD1 homologous recombination function is also required to control the levels of viral genome replication. Future studies will focus on determining what structural and enzymatic functions of SAMHD1 contribute toward the control of HPV16-induced cellular proliferation and what viral proteins SAMHD1 interacts with to regulate this control. Ultimately, it may be possible to engineer elevated levels of functional SAMHD1 in the presence of HPV16 that could block HPV16-induced cellular proliferation and amplification of the viral genome.

## MATERIALS AND METHODS

### Cell culture.

Clonal cell lines containing the HPV16 genome were generated from N/Tert-1 cells, as previously described (7). These cells were cultured alongside parental N/Tert-1 cells for all comparisons. N/Tert-1 and N/Tert-1+HPV16 cells were grown in keratinocyte serum-free medium (K-SFM; Invitrogen) with a 1% (vol/vol) penicillin-streptomycin mixture (Thermo Fisher Scientific) containing 4 μg/ml hygromycin B (Millipore Sigma) at 37°C in a 5% CO_2_–95% air atmosphere and passaged every 3 to 4 days. N/Tert-1+HPV16 cells were grown in the same medium also containing 150 μg/ml G418 (Thermo Fisher Scientific). HTK+HPV16-CRISPR guides to SAMHD1 were delivered into N/Tert-1, N/Tert-1+HPV16, and HTK+HPV16 via lentivirus, and cells were selected by growth in puromycin containing K-SFM (2 μg/ml; Millipore Sigma). All cells were routinely checked for mycoplasma contamination. For downstream protein and RNA analyses, 1 × 10^6^ cells were plated onto 100-mm plates, trypsinized, pelleted after 24 h, and washed twice with phosphate-buffered saline (PBS).

### SAMHD1 depletion by CRISPR/Cas9 genome editing.

CRISPR/Cas9-mediated SAMHD1 depletion was described previously (25). Briefly, three different single guide RNAs (sgRNAs) targeting human SAMHD1 were designed and cloned into lentiCRISPR v2 vector (Addgene plasmid no. 52961). Packaging 293T cells were transfected with SAMHD1 sgRNAs (CRISPR SAMHD1) or a nontargeting sgRNA control and helper vectors (pMD2.G and psPAX2; Addgene plasmid numbers 12259 and 12260, respectively) using Lipofectamine 2000 reagent (catalog no. 11668019; Life Technologies). Medium containing lentiviral particles and 8 mg/ml Polybrene (Sigma-Aldrich, St. Louis, MO) was used to infect N/Tert-1 or HTK+HPV16 cells. Infected cells were selected in medium containing 2 μg/ml puromycin. The target guides sequences are as follows: for SAMHD1-sg1, forward (F), 5′-CACCGCTTAGTTATATCCAGCGAT-3′; and reverse (R), 5′-AAACATCGCTGGATATAACTAAGC-3′; for SAMHD1-sg2, F, 5′-CACCGAATCCACGTTGATACAATGA-3′; and R, 5′-AAACTCATTGTATCAACGTGGATTC-3′; for SAMHD1-sg3, F, 5′-CACCCGTCTTCGATACATCAAACAGC-3′; and R, 5′-AAACGCTGTTTGATGTATCGAAGAC-3′; and for sgRNA-control, F, 5′-CACCGTTCCTAAGATTTTTAAGACT-3′; and R, 5′-AAACAGTCTTAAAAATCTTAGGAAC-3′.

### qPCR.

qPCR was performed on 10 ng of DNA Hirt extracted from monolayer or organotypic raft grown cells. DNA and relevant primers were added to PowerUp SYBR green master mix (Applied Biosystems) and real-time PCR performed using the 7500 Fast real-time PCR system, using SYBR green reagent. Primer sequences were HPV16 E2 F, 5′-ATGGAGACTCTTTGCCAACG-3′; HPV16 E2 R, 5′-TCATATAGACATAAATCCAG-3′; HPV16 E6 F, 5′-TTGAACCGAAACCGGTTAGT-3′; and HPV16 E6 R, 5′-GCATAAATCCCGAAAAGCAA-3′. As HIRT buffer is optimized for the isolation of small DNA, mitochondrial DNA was detected as the internal control; the primers were F, 5′-caggagtaggagagagggaggtaag-3′; and R, 5′-tacccatcataatcggaggctttgg-3′.

### SYBR green qRT-PCR.

RNA was isolated using the SV Total RNA isolation system (Promega), following the manufacturer’s instructions. Two micrograms of RNA was reverse transcribed into cDNA using the high-capacity reverse transcription kit (Applied Biosystems). cDNA and relevant primers were added to PowerUp SYBR green master mix (Applied Biosystems) and real-time PCR performed using 7500 Fast real-time PCR system. The primer sequences were SAMHD1 F, 5′-ctggaactccatcccgactac-3′; SAMHD1 R, 5′-agtaatgcgcctgtgatttcat-3′; glyceraldehyde-3-phosphate dehydrogenase (GAPDH) F, 5′-ggagcgagatccctccaaaat-3′; GAPDH R, 5′-ggctgttgtcatacttctcatgg-3′.

### Protein analysis.

Cells (1 × 10^6^) were lysed in 50 μl NP-40 lysis buffer (0.5% Nonidet P-40, 50 mM Tris [pH 7.8], 150 mM NaCl) supplemented with protease inhibitor (Roche Molecular Biochemicals) and phosphatase inhibitor cocktail (Sigma). The cell and lysis buffer mixture was incubated on ice for 20 min and centrifuged for 20 min at 184,000 relative centrifugal force (rcf) at 4°C, and supernatant was collected. Protein levels were determined utilizing the Bio-Rad protein estimation assay. Equal amounts of protein were boiled in 2× Laemmli sample buffer (Bio-Rad). Samples were then loaded into a Novex 4 to 12% gradient Tris-glycine gel (Invitrogen), run at 100 V for approximately 2 h, and then transferred onto nitrocellulose membranes (Bio-Rad) at 30 V overnight using the wet blot method. Membranes were blocked in Odyssey blocking buffer (diluted 1:1 with PBS) at room temperature for 6 h and probed with relevant antibody diluted in Odyssey blocking buffer overnight at 4°C. Membranes were then washed with PBS supplemented with 0.1% Tween (PBS-Tween) before probing with corresponding Odyssey secondary antibody (goat anti-mouse IRdye800cw or goat anti-rabbit IRdye680cw) diluted 1:10,000 for 1 h at 4°C. Membranes were washed in PBS-Tween before infrared scanning using the Odyssey CLx Li-Cor imaging system. The following antibodies were used for Western blot analysis: GAPDH (1:10,000; Santa Cruz sc-47724), SAMHD1 (1:1,000; Cell Signaling Technology), and V5 (1:500; Abcam).

### Organotypic raft culture.

N/Tert-1, N/Tert-1+HPV16, and HTK+HPV16 cells were differentiated via organotypic raft culture, as described previously (46, 47). Briefly, cells were seeded onto type 1 collagen matrices containing J2 3T3 fibroblast feeder cells. Cells were then grown to confluence on top of the collagen matrices, which were then lifted onto wire grids and cultured in cell culture dishes at the air-liquid interface, with medium replacement on alternate days. Following 13 days of culture, rafted samples were fixed with formaldehyde (4% [vol/vol]) and embedded in paraffin blocks. Multiple 4-μm sections were cut from each sample. Sections were stained with hematoxylin and eosin (H&E) and others prepared for immunofluorescent staining, as described previously.

### Immunofluorescence.

The antibodies used and relevant dilutions are as follows: SAMHD1, 1:1,000 (Cell Signaling Technology); BrdU, 1:200 (Cell Signaling Technology); cyclin E, 1:1,000 (Sigma-Aldrich); and V5, 1:500 (Abcam). Immune complexes were visualized using Alexa 488- or Alexa 595-labeled anti-species-specific antibody conjugates (Molecular Probes). Cellular DNA was stained with 4′,6-diamidino-2-phenylindole (DAPI; Santa Cruz sc-3598). Fluorescent *in situ* hybridization (FISH) staining for HPV16 genomes was performed using digoxigenin (DIG)-labeled HPV16 genomes, as described previously (48, 49). Immunofluorescence was quantified, using a Vectra Polaris automated imaging system, whereby whole stained sections were scanned computationally and the intensity calculated compared to a negative background control (secondary antibody only) and a positive localization control (DAPI). The same imaging parameters were used for each slide. For each sample, two sections from three individual rafts were scanned to generate average values. Immunofluorescence was observed using a LSM 710 laser scanning microscope and Zen 2011 software (Carl Zeiss). Images were assembled in Adobe Photoshop CS 6.0.

### Southern blotting.

Total cellular DNA was extracted using a phenol-chloroform method and 5 μg digested with SphI to linearize the HPV16 genome. All digests included DpnI to ensure that all input DNA was digested and not represented as replicating viral DNA. Digested DNA was separated by electrophoresis of a 0.8% agarose gel, transferred to a nitrocellulose membrane, and probed with radiolabeled (32-P) HPV16 genome. This was then visualized by exposure to film for 24 or 72 h.

### Statistics.

Standard error was calculated from three independent experiments and significance determined using a Student’s *t* test.
