# Supplementary figures and images for: SAMHD1 Regulates Human Papillomavirus 16-Induced Cell Proliferation and Viral Replication during Differentiation of Keratinocytes
Source: mSphere. 2019 Aug 7;4(4):e00448-19. doi: 10.1128/mSphere.00448-19 (PMC6686230; doi:10.1128/mSphere.00448-19)

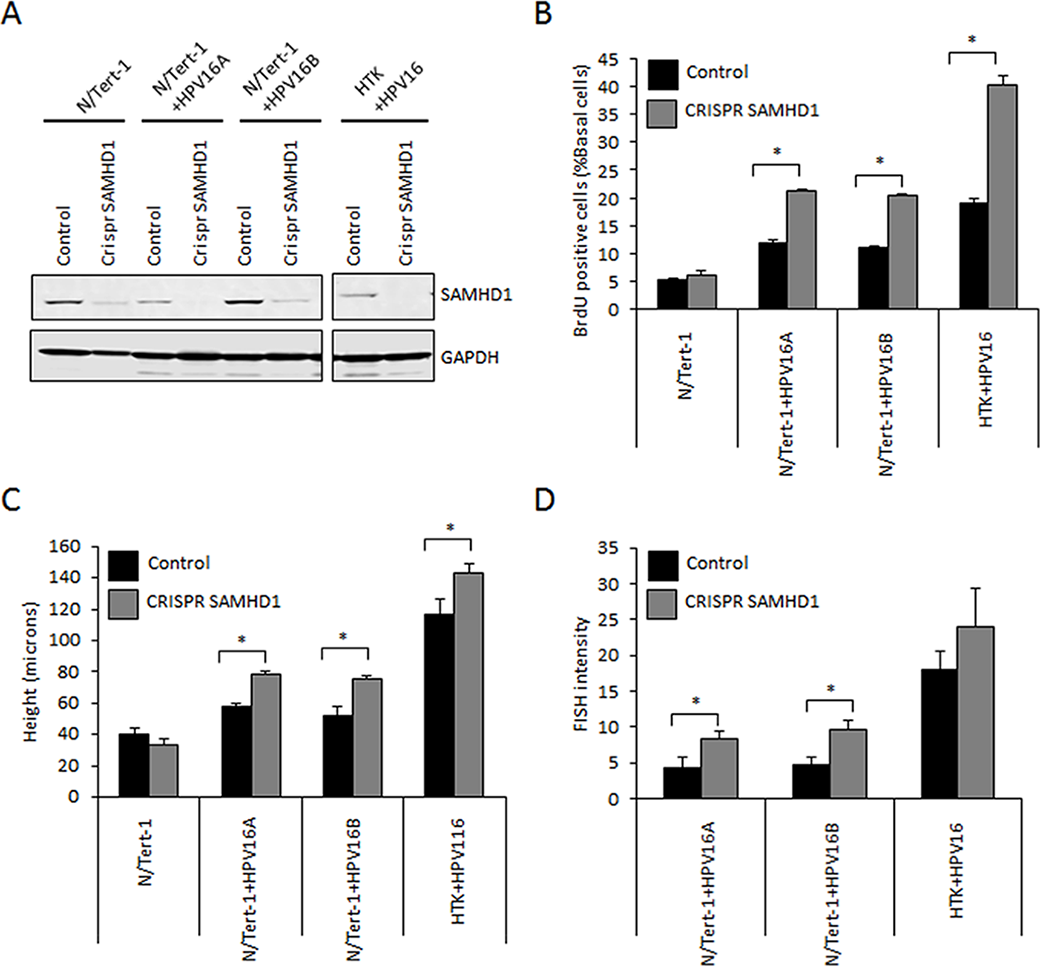

Supplement: FIG S1 [file mSphere.00448-19-sf001.tif]
